# The Role of Virtual Reality in Chronic Pain and Loneliness: Narrative Review

**DOI:** 10.1089/jmedxr.2024.0063

**Published:** 2025-07-07

**Authors:** Alicia Pan, Sacha Moreau, Ghazal Rafeeh, Naba Ahsan, Christopher L. Robinson, Min Lang, Margot Paul, James Khan, Alexandra Thérond

**Affiliations:** ^1^ Department of Mental Health, Johns Hopkins Bloomberg School of Public Health, Baltimore, Maryland, USA.; ^2^ Department of Architecture, Massachusetts Institute of Technology, Boston, Massachusetts, USA.; ^3^ Department of Biomedical and Molecular Sciences, Queen’s University, Kingston, Canada.; ^4^ Department of Cognitive Science, Carleton University, Ottawa, Canada.; ^5^ Department of Anesthesiology, Perioperative, and Pain Medicine, Harvard Medical School, Brigham and Women’s Hospital, Boston, Massachusetts, USA.; ^6^ Department of Radiology, Harvard Medical School, , Boston, Massachusetts, USA.; ^7^ Department of Psychiatry and Behavioral Sciences, Stanford University School of Medicine, Stanford, California, USA.; ^8^ Department of Anesthesiology and Pain Medicine, University of Toronto, Toronto, Canada.; ^9^ Department of Psychology, Université du Québec à Montréal, Montréal, Canada.

**Keywords:** immersive technology, pain management, machine learning, loneliness, social isolation, chronic pain

## Abstract

Chronic pain and loneliness are significant public health challenges that often intersect, leading to a cyclical cycle of physical, emotional, and social distress. The COVID-19 pandemic has further exacerbated these issues, highlighting the need for innovative solutions. Virtual reality (VR) has emerged as a promising tool for managing chronic pain and alleviating loneliness, but its potential to address the complex interplay between these conditions remains underexplored. This narrative review aims to synthesize current evidence on the effectiveness of VR interventions in managing chronic pain and loneliness, identify key themes and knowledge gaps, and provide recommendations for future research and clinical applications. A literature search was conducted, focusing on randomized controlled trials, cohort studies, and case–control studies published between January 2020 and December 2024 that investigated VR interventions for chronic pain and loneliness in adult populations. Studies were analyzed for recurring themes, trends, applications, and implications. VR interventions demonstrate the potential to reduce pain intensity, improve mood, and foster social connectedness through immersive, interactive experiences. Several key themes emerged, including the importance of multisensory stimulation, personalization, and social presence in enhancing VR’s therapeutic effects. However, challenges related to accessibility, user experience, and long-term efficacy remain. VR offers a promising approach to managing chronic pain and loneliness, with the potential to improve patient outcomes and quality of life. Future research should address identified barriers, develop culturally adaptive interventions, leverage artificial intelligence for personalization, and conduct longitudinal studies to assess long-term benefits and potential side effects. Integrating VR into collaborative health care teams and comprehensive treatment plans has the potential to maximize its impact and help with cost efficiencies. Advancing VR technology will require continued research to maximize its potential to enhance physical, emotional, and social well-being for those experiencing chronic pain and loneliness.

## Introduction

Loneliness is a significant issue among older adults aged 65 and above, affecting approximately one in four individuals in this population.^1^ Loneliness, a subjective and distressing experience characterized by a perceived lack of social connections or a sense of belonging,^2^ often intersects significantly with chronic pain. Loneliness often exacerbates health conditions, such as chronic pain. Chronic pain is defined as “an unpleasant sensory or emotional experience associated with, or resembling that associated with, actual or potential tissue damage,” persisting for longer than 3 months. Research shows that, on average, one-third of older adults report experiencing pain that persists for at least 6 months, and one in five report moderate to severe pain within the past 3 months.^3–5^ Chronic pain, much like loneliness, reduces quality of life and poses critical challenges to physical and emotional well-being.

Chronic pain constitutes a significant public health challenge, which contributes to sustained physical and psychological distress.^6^ It is frequently accompanied by physical impairments and considerable mental health comorbidities, such as anxiety, depression, and social isolation, which can further worsen the pain’s severity and persistence.^7^ These interrelated conditions complicate the diagnostic process and the development of effective treatment strategies. Therefore, comprehensive, multidisciplinary interventions that address both the physiological dimensions of pain and its psychological consequences are essential to mitigate the risk of further emotional, cognitive, and social decline.

Emerging research indicates a significant association between loneliness and chronic pain, though the direction of this relationship remains unclear. Some studies suggest that chronic pain can lead to social withdrawal and exacerbate feelings of loneliness due to limitations on mobility and social participation.^8^ Conversely, other research indicates that loneliness may magnify the perception of pain, potentially contributing to the development or worsening of chronic pain conditions.^9^ Furthermore, neuroimaging studies have identified common brain mechanisms underlying physical and social pain. Functional magnetic resonance imaging research has further shown that experiences of social rejection or isolation activate brain regions associated with physical pain processing, such as the anterior cingulate cortex and insula.^10,11^ These shared neural pathways suggest that social and physical pain are processed similarly in the brain, providing a neurobiological basis for the interplay between loneliness and chronic pain.

Addressing the interconnected challenges of loneliness and chronic pain requires approaches that go beyond traditional treatments. The biopsychosocial model, which acknowledges the complex interplay between biological, psychological, and social factors in health and illness, offers a valuable framework for understanding how these issues are related and how they might be addressed.^12^ One promising avenue is the use of virtual reality (VR) as an intervention.

VR has emerged as a health care tool, with applications ranging from physical rehabilitation to mental health support. It offers immersive, interactive environments designed to address specific health challenges, rendering it an appealing option for older adults who may struggle with physical limitations and feelings of isolation.^13^ VR interventions can engage users, distract from pain, and foster social connections, even without in-person interaction.^14,15^

This narrative review examines the growing body of evidence supporting the use of VR in alleviating symptoms of both chronic pain and loneliness. By exploring current research, we can aim to gain a deeper understanding of VR’s potential role in improving the lives of individuals managing chronic conditions. As loneliness and chronic pain continue to impact many individuals, exploring innovative, evidence-based solutions like VR that target the biopsychosocial aspects of health becomes increasingly important.

## Methods

The review followed the methodological guidelines outlined by Popay et al. for conducting systematic narrative reviews.^16^ The aim of this article was to explore the intersection of VR interventions, chronic pain, and loneliness, focusing on the therapeutic and social benefits of VR in managing chronic pain and alleviating loneliness. This article further aims to highlight the rapid development of VR technologies and the impact of the COVID-19 pandemic on health care and social isolation.

A systematic literature search was conducted using PubMed and Google Scholar. Search terms included “loneliness,” “environmental factors,” “chronic pain,” “virtual reality,” and “digital health.” We focused on studies from 2020 to 2024 as this period saw increased interest in those topics as well as a significant shift in VR research and technology. The period is marked by the U.S. surgeon general’s focus on loneliness, social isolation, and its influence on chronic health issues, as well as the emergence of consumer-grade stand-alone headsets like the Quest 2, improved software development frameworks, and notably, over half of all PubMed-indexed VR studies were published during the 2020–2024 period. The review included randomized controlled trials (RCTs), cohort studies, and case–control studies published between 2020 and 2024, and included earlier studies when necessary for context or comparison. We included articles published in English emphasizing recent evidence that investigated VR interventions related to chronic pain, loneliness, or environmental influences on these conditions, focusing on adult populations (18+). We excluded studies that involved pediatric populations, nonhuman subjects, qualitative research methodologies, or diseases unrelated to chronic pain or loneliness. Data extraction was performed using a structured Excel-based form developed by the authors. Two reviewers (A.T. and A.P.) independently conducted the screening and data extraction to ensure accuracy and reliability. Any disagreements were resolved through discussion, with the involvement of a third reviewer (S.M.) when necessary.

## Results

**FIG. 1.**
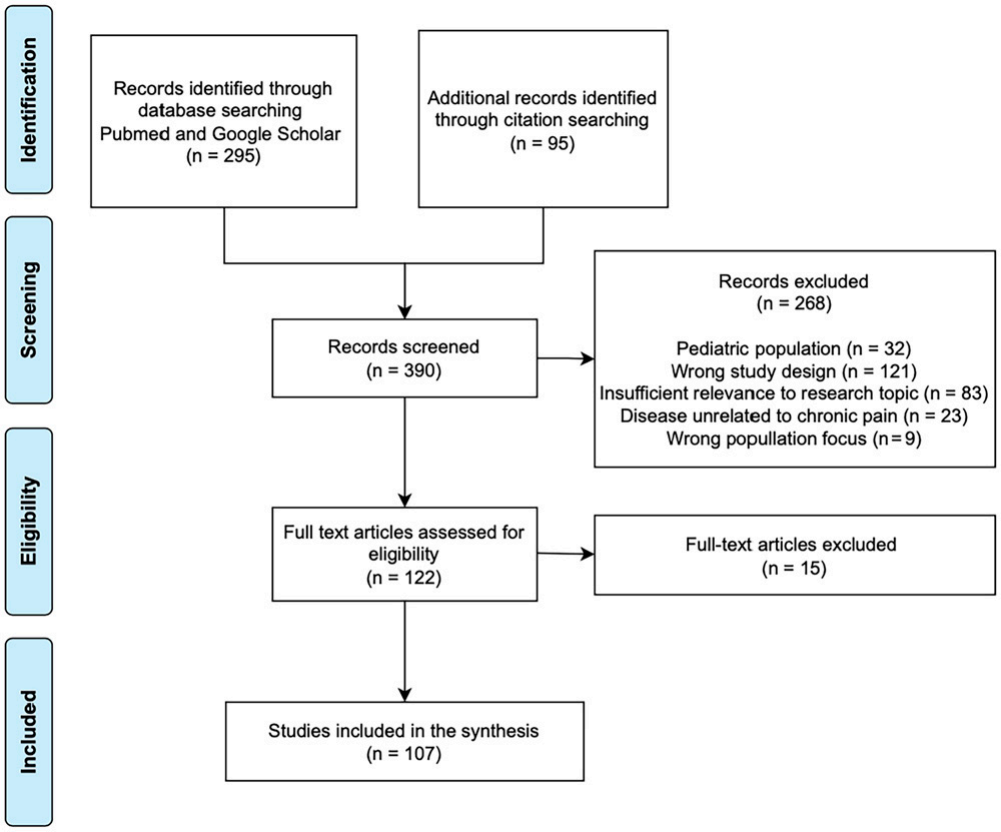
Flowchart illustrating the literature search and study selection procedure. (Figure created by A.T.).

The search yielded 280 articles as shown in Figure 1. After removing duplicates, the titles and abstracts of the remaining records were screened for relevance. To ensure thoroughness, additional literature was identified through hand-searching the reference lists of included studies. This process retrieved 95 further articles that were not captured in the initial search, expanding the scope of the review and providing a more comprehensive view of the field.

### Consequences of loneliness and chronic pain exacerbated by social isolation

**FIG. 2.**
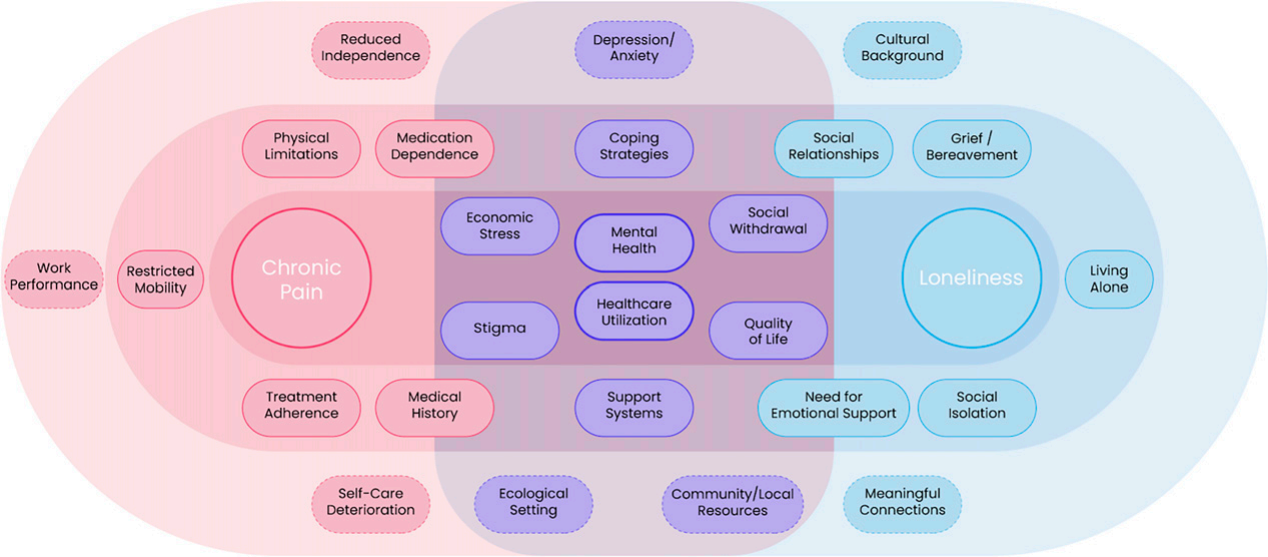
Intersection of chronic pain and loneliness, focusing on various psychosocial determinants of health and its consequences. (Image created by S.M.).

Understanding the intersection between chronic pain and loneliness is fundamental, as chronic pain and loneliness can mutually intensify a cycle of worsening health and social withdrawal, as displayed in Figure 2. Social isolation often accompanies chronic pain, amplifying its effects. Additionally, social determinants of health, such as living alone, experiencing bereavement, and lacking meaningful social connections, as illustrated in Figure 2, can contribute to the development of loneliness. Financial difficulties, from reduced work capacity, avoidance behaviors developed as adaptations to pain, and physical limitations that prevent participation in social activities all contribute to a narrowing of social experiences and heightened feelings of loneliness.^17^ The COVID-19 pandemic and enforced social distancing further highlighted this intersection, with individuals with chronic pain experiencing an increase in loneliness due to reduced social interactions and support.^18^

Longitudinal studies have shown that these effects have persisted beyond the pandemic’s acute phase, with chronic pain patients reporting sustained increases in both pain intensity and psychological distress compared with prepandemic levels.^19^ For many with chronic pain, the onset of social distancing led to increased pain interference and elevated feelings of loneliness.^18^ This period also exacerbated specific conditions, such as chronic low back pain,^20^ and increased levels of pain catastrophizing, a cognitive process where patients anticipate pain-related experiences more intensely.^18^ Additionally, there was a perceived increase in pain severity compared with presocial distancing levels.^21^

Chronic pain further isolates individuals by making their struggles less visible to those around them. Thus, loneliness can be viewed as subjectively painful suffering from the absence of social connections or a lack of belonging, a perception reflected through the discrepancy between social needs and their availability in the environment.^22^ For individuals with chronic pain, this understanding is often intensified, leading to a self-perpetuating cycle where loneliness exacerbates chronic pain and vice versa.^23^ Addressing this intersection is vital for developing more holistic health interventions that target both physical symptoms and the underlying social and emotional factors contributing to suffering.

### Ecological drivers of loneliness

Loneliness and chronic pain combined form a vicious cycle of physical and mental health challenges, each exacerbating the other as highlighted in Figure 2.^24–26^ The COVID-19 pandemic has further worsened this issue, creating barriers to social connection and contributing to a deterioration in mental health. Figure 2 further highlights the central role of mental health in both chronic pain and loneliness, suggesting its influence in both the deterioration and healing processes of these conditions. This includes an increase in loneliness, social isolation, anxiety, and depression.^27^ Individuals with these “invisible illnesses” are at a higher risk of receiving lower social support and experiencing feelings of invisibility, which can lead to loneliness and isolation.^28^ In chronic pain cases, social isolation often arises due to symptoms such as anxiety from the apprehension of pain “flare-ups,” fatigue due to the body fighting pain, and brain fog or temporary cognitive impairment.^29^ This isolation creates more opportunities to contemplate and engage in negative thinking about pain, ultimately exacerbating the pain and entering a vicious pain–loneliness cycle.^30^

Previous research has indicated that rural residents often experience poorer mental health outcomes associated with loneliness.^31,32^ While it was initially hypothesized that urban and rural settings would present different challenges during pandemic-related social distancing, subsequent research by Henning-Smith challenged this assumption.^33^ Their study found that among older adults experiencing enforced social isolation during the pandemic, there was no discernible difference in feelings of loneliness between those living in rural versus urban environments, suggesting that the impact of social distancing transcended geographical settings.

Moreover, research suggests that a lack of autonomy over environmental changes has been linked to heightened loneliness.^34^ Social inclusivity, engaging with community activities, engaging with nature, engaging among cohesive neighborhoods, and physical activity are all known to reduce loneliness.^35–38^ As shown in Figure 2, factors, such as quality of life, social withdrawal, community and local resources, and support systems significantly influence an individual’s ability to guard against loneliness. In inadequate environments, these factors may instead act as barriers, making it harder to prevent or cope with loneliness. Abrupt environmental changes can hinder social interactions, making it challenging to form meaningful connections and engage in social pursuits, consequently amplifying loneliness.^39^ Understanding social engagement’s role in an individual’s community and environment is crucial in understanding the connection between environmental factors and loneliness.

### Active components of VR for loneliness, social isolation, and chronic pain

**FIG. 3.**
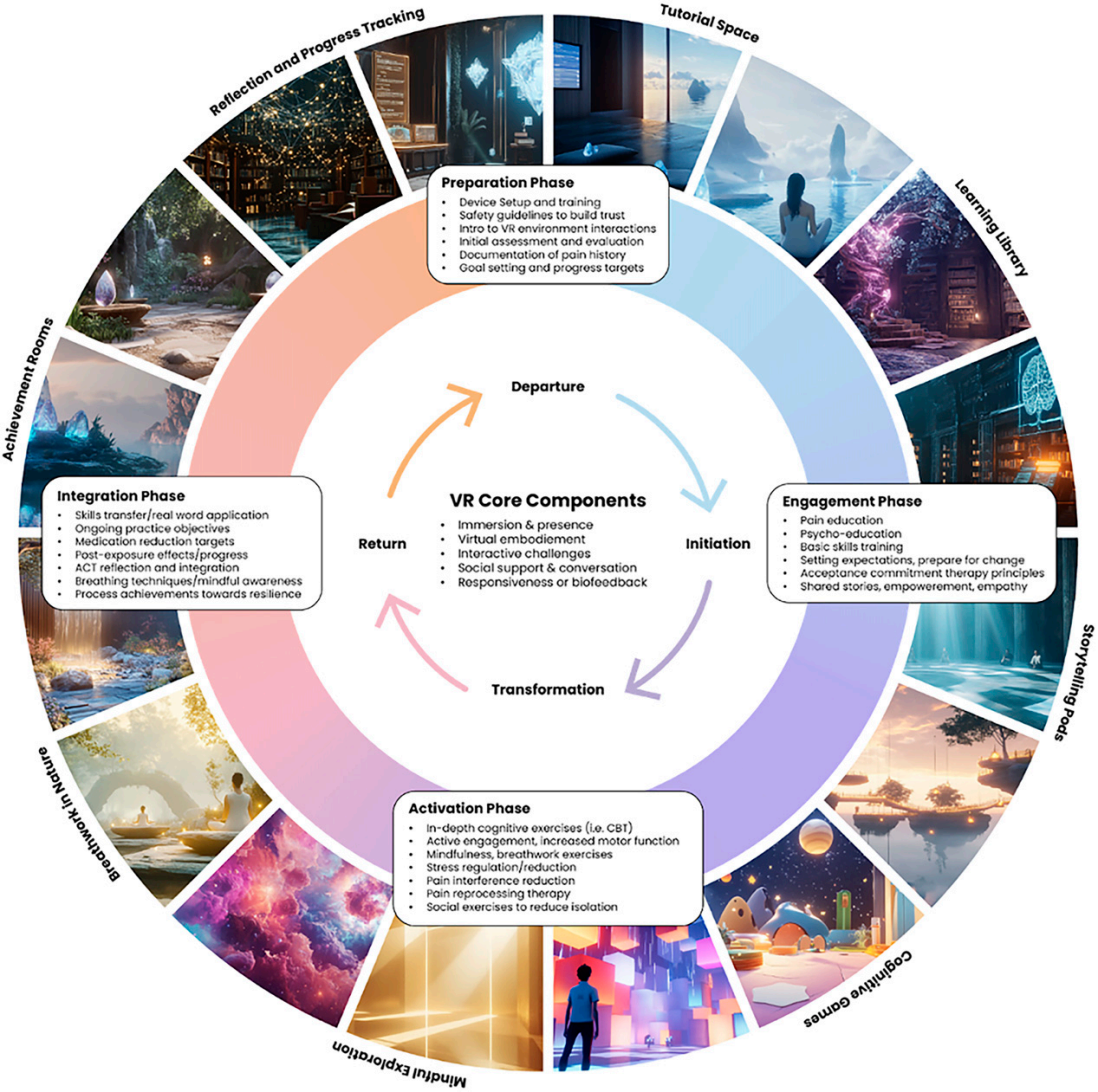
Example of core components for the effective development and design of VR environments and experiences across individual modules and therapies. (Image created by S.M.).

VR offers a promising intervention to address the intersection of loneliness and chronic pain. By creating immersive and interactive environments, VR can replicate the benefits of natural and social settings that are otherwise inaccessible due to living conditions or physical limitations. As Figure 3 depicts, the transition into VR to create an impactful experience that leads to high engagement, immersion, and retention from users requires a staged approach with varied levels of interaction. For example, a study that used the multimodal Ripple experience (Table 1) involving 38 women with metastatic breast cancer using two different weeklong VR interventions showed significant and sustained improvements across several domains including quality of life (mean increase of 0.06 on the EuroQol 5-Dimension 5-Level (EQ-5D-5L) index, *p* < 0.001), fatigue (mean reduction of 5.00 on the Functional Assessment of Chronic Illness Therapy (FACIT)-Fatigue scale, *p* < 0.001), depression (mean reduction of 3.69 points, *p* < 0.001), and stress (mean reduction of 3.07 points, *p* < 0.001).^40^

**Table 1.** List and Descriptions of Virtual Reality Softwares Utilized by Referenced Studies

VR software name	Reference(s)	Modality type	Software description	Environment design type	Hardware used
Happy Place	Reynolds et al. (2022)^40^	Relaxation with interactive elements	Tranquil/animated camping scene with changes to weather and time of day. Includes optional interactive tasks, guided relaxation, and soothing music. Developed by Hj√§rtat in 2016.	3D-generated environment	Pico Goblin VR headset with controller and Panasonic RP-HT161 headphones
Ripple	Reynolds et al. (2022)^40^	Interactive relaxation	Collection of three short 360 VR nature scenes developed by Mixt Studio: (1) a beach where participants can write words in sand/sky, (2) a waterfall where participants can stack stones, and (3) a mountain range where participants can jump between different locations.	360 image with interactive elements	Pico Goblin VR headset with controller and Panasonic RP-HT161 headphones
Virtual Iceland	Garrett et al. (2017)^41^	Passive VR experience	Virtual environment of Iceland that users passively travel through/part of a set of passive VR experiences used in week 1 of the study.	3D-generated environment	Oculus Rift DK2 with high-end PC
Senza Peso	Garrett et al. (2017)^41^	Passive VR experience	Artistic boat ride experience/part of a set of passive VR experiences used in week 1 of the study.	3D-generated environment	Oculus Rift DK2 with high-end PC
3D Mandalas	Garrett et al. (2017)^41^	Mindfulness and meditative	Experience involving flying through 3D mandalas/used in week 2 of the study as part of mindfulness and meditative-focused applications.	3D-generated environment	Oculus Rift DK2 with high-end PC
Sightline	Garrett et al. (2017)^41^	Mindfulness and meditative	Experiences that alter the user’s environment depending on where they looked/used in week 2 of the study.	Interactive 3D-generated environment	Oculus Rift DK2 with high-end PC
Underwater Environment	Garrett et al. (2017)^41^	Active exploratory	Environment allowing exploration of underwater scenes/used in week 3 of the study.	Interactive 3D-generated environment	Oculus Rift DK2 with high-end PC
Solar System	Garrett et al. (2017)^41^	Active exploratory	Environment allowing exploration of the solar system/used in week 3 of the study.	Interactive 3D-generated environment	Oculus Rift DK2 with high-end PC
Natural Environment	Garrett et al. (2017)^41^	Active exploratory	Environment allowing exploration of a natural setting/used in week 3 of the study.	Interactive 3D-generated environment	Oculus Rift DK2 with high-end PC
3D Puzzle Games	Garrett et al. (2017)^41^	Active problem solving	Game-type environments requiring participants to solve 3D puzzles/used in week 4 of the study.	Interactive 3D generated environment	Oculus Rift DK2 with high-end PC
Reducept	Groenveld et al. (2023)^42^	Gamified interaction with education	Designed as a game where patients “travel” through the nervous system in a pod. A calm voice explains pain mechanisms with visual representations. Includes five different games based on psychological treatment principles (acceptance and commitment therapy, mindfulness, hypnotherapy, eye movement, and desensitization and reprocessing) with educational elements about nervous system changes.	Interactive 3D-generated environment	Oculus Go head-mounted display
AppliedVR Pain Management	Darnall et al. (2020)^43^	Multimodal (CBT skills, relaxation, mindfulness)	Self-management skills program based on CBT principles with biofeedback and mindfulness. Three main content categories: (1) pain CBT with didactics on thought restructuring, (2) relaxation training with diaphragmatic breathing and visual biofeedback responding to users’ breathing, and (3) mindfulness for body awareness and thought release.	Interactive 3D-generated environment with biofeedback	Oculus Go VR headset
UE4 Virtual Reality Simulation	Rogers et al. (2022)^46^	Social interaction	Basic virtual room where VR interactions took place. The participant was positioned on one side of a desk facing a realistic avatar controlled by motion capture of a real person. The avatar mimicked the face and body movements of a real person in real time.	Interactive 3D-generated environment with motion-captured avatars	Oculus Rift S connected to high-end PC with Perception Neuron motion capture system and iPhone facial tracking
N/A (Custom 360 Videos)	Brown (2019)^47^	Passive viewing/distraction	Six custom 360 videos ranging from 30 s to 3 min, showing familiar local environments, including walks through art museum, hiking trail, church sanctuary, and driving scenes through local neighborhood, main street, and historic landmark grounds.	360 videos	Samsung Gear VR headset with Samsung smartphone and handheld controller
EaseVRx (aka RelieveVRx)	Maddox et al. (2023)^48^, Garcia et al. (2021)^49^, Maddox et al. (2024)^50^, Colloca et al. (2025)^67^, Spiegel et al. (2019)^81^	Multimodal (skills-based pain management)	Fixed sequence of 56 VR experiences incorporating evidence-based principles of CBT, mindfulness, and pain neuroscience education. Content categories include (1) pain education (visual and voice-guided lessons), (2) relaxation/interoception (progressive scenes), (3) mindful escapes (360 videos with therapeutic voiceovers, music, guided breathing), (4) pain distraction games, and (5) dynamic breathing (biofeedback training in interactive environments). In the Colloca study, 10 preselected relaxing experiences were used featuring ambient/soothing music with guided relaxation and naturalistic scenes.	Mixed modalities (360 videos/interactive 3D environments with biofeedback)	Pico G2 4K all-in-one head-mounted VR device (Garcia et al., 2021); Samsung Gear Oculus headset with Samsung Galaxy S7 phone (Spiegel et al., 2019); device not specified in other studies
Fulldive VR	Morales Tejera et al. (2020)^53^	Distraction with therapeutic exercise	Simulates the living room of a house with a gallery of selected photos. Users change images in the viewfinder by tilting their neck bilaterally, providing a first degree of difficulty exercise requiring only lateral neck movements.	3D-generated environment	VR Vox Play glasses with HMD clamping system and LG Q6 smartphone
VR Ocean Aquarium 3D	Morales Tejera et al. (2020)^53^	Distraction with therapeutic exercise	Virtual environment simulating an ocean where users move forward to observe different marine animals by making neck movements (flexion, extension, rotation). Includes auditory and sensory stimuli with sound of the sea.	3D-generated environment with sensory integration	VR Vox Play glasses with HMD clamping system and LG Q6 smartphone
SnowCanyon	Hoffman et al. (2024)^68^	Distraction with interactive elements	3D computer-generated snowy canyon where participants float along a predefined path. In the fully immersive condition, participants could interact with objects (e.g., throw snowballs at snowmen and penguins using head movements/gaze direction).	Interactive 3D-generated environment	HTC VIVE Pro VR helmet with base stations
BearBlast	Gold et al. (2024)^69^	Distraction with gamified interaction	Multisensory (visual and auditory) VR game where users travel on a preset path through a colorful and immersive 3D environment with animated landscapes, buildings, and clouds. Users’ gaze controls the direction of a cannon fired to knock down teddy bears.	Interactive 3D-generated environment	Oculus Gear VR with head-tracking system
N/A (YouTube 360 Videos)	Burrai et al. (2023)^70^	Passive distraction/relaxation	310 videos in nine categories: (1) Africa, (2) hills, (3) rivers, lakes, and waterfalls, (4) islands, (5) deserts, (6) beaches, (7) mountains, (8) sea, and (9) submarines. Videos featured background nature sounds and soothing musical stimuli in high-definition panning stereo. Videos were described as natural environments to activate powerful distraction and positive relaxation.	360 spherical video	Oculus Quest 2 HMD with joystick for navigation and earphones
Figmin XR with AI voice cloning	Jumreornvong et al. (2024)^73^	Personalized relaxation/multimodal	Interactive VR environments enhanced by AI-generated voice narration that replicates familiar individuals’ voices using Eleven-Labs’ voice cloning (Tacotron and WaveNet). Features relaxing island settings with elements such as flowers, trees, and animals. The environment was initially generated using DALL-E with 3D models created using Meshy AI 3D and Sloyd. The AI voices guide users through the VR environment, supporting emotional regulation and relaxation.	AI-generated interactive 3D environments	HMD (specific model not mentioned)
N/A (Natural Environments)	Wiederhold et al. (2014)^74^	Relaxation with guided breathing	Pleasant and relaxing scenes including natural areas such as forests, beaches, and mountains with relaxing music. Included soothing effects such as branches swaying and tall grass moving (six to eight times per minute) specifically designed to guide breathing regulation.	3D-generated environment	HMD (specific model not mentioned)
Altspace VR	Kenyon et al. (2023)^85^	Social interaction	Social VR platform with shared virtual spaces where users can interact with each other in a research room environment.	3D-generated environment for social interaction	Accessible on Oculus Quest/Oculus GO/Oculus Rift/HTC Vive/PC
eXtended-reality Artificial Intelligence Assistant (XAIA)	Spiegel et al. (2024)^87^	Multimodal (therapeutic conversation with AI)	VR application merging VR and AI to offer personalized/CBT-based mental health support. Users select from nine nature scenes and interact with “XAIA,” a robot avatar. Conversations are processed through a HIPAA-compliant server: audio is recorded, transcribed by speech-to-text AI, and responded to by LLM (GPT-4) with an “Appropriateness Classifier” to filter potentially dangerous responses. System applies psychotherapeutic techniques including reflection, empathy, validation, and cognitive restructuring.	3D-generated environment with AI interaction	Meta Quest 2
N/A (Vizard VR Environments)	Pot-Kolder et al. (2018)^89^	Cognitive behavioral therapy	Four virtual social environments (street, bus, cafe, and supermarket) created with Vizard software. Therapists could control various elements, including number of avatars (0–40), their characteristics (sex/ethnicity), and their responses to the patient (neutral or hostile, eye contact), creating personalized treatment exercises.	Interactive 3D-generated environment	Sony HMZ-T1/T2/T3 Head-Mounted Display with Logitech F310 Gamepad
Ocean Rift	Frey et al. (2019)^93^	Distraction	Scene of curious manatees from Ocean Rift scuba diving simulation with sounds of manatee calls and underwater breathing. Additional relaxing music from Brain.fm. User input included head tracking and a hand control that simulated taking underwater photos.	Interactive 3D-generated environment	Samsung GearVR head-mounted display with Galaxy S7 phone/hand control/and noise-reducing headphones
Paint Studio	Mosadeghi et al. (2016)^97^	Interactive distraction	VR application where users “paint” a picture using head gestures to control the paintbrush.	Interactive 3D-generated environment	Samsung Gear VR Innovator edition with Samsung Galaxy Note 4
TheBluVR	Mosadeghi et al. (2016)^97^	Passive distraction	Underwater ocean exploration experience.	3D-generated environment	Samsung Gear VR Innovator edition with Samsung Galaxy Note 4
Cirque du Soleil	Mosadeghi et al. (2016)^97^	Passive distraction	Experience where users share the stage with performers performing aerial acrobatics while suspended from long/silk bands of fabric.	360 video	Samsung Gear VR Innovator edition with Samsung Galaxy Note 4
Tours of Iceland	Mosadeghi et al. (2016)^97^	Passive distraction	Aerial tour of rich Icelandic topographies.	360 video	Samsung Gear VR Innovator edition with Samsung Galaxy Note 4
Pain RelieVR	Tashjian et al. (2017)^100^	Distraction with gamified interaction	15-min immersive/360° game experience in a fantasy world where users attempt to shoot balls at moving objects by maneuvering their head toward targets. Nonviolent, noncompetitive game with motivational music, positively reinforcing sounds, animation, and direct messages. Forward-facing action designed for bedbound patients.	Interactive 3D-generated environment	Samsung Gear Oculus headset with Samsung Galaxy S7 phone

AI, artificial intelligence; CBT, cognitive behavioral therapy; GPT-4, Generative Pre-trained Transformer 4; HIPAA, Health Insurance Portability and Accountability Act; HMD, head-mounted display; LLM, large language model; VR, virtual reality.

A meta-analysis and systematic review assessed the effectiveness of VR-based interventions for managing cancer-related symptoms such as anxiety, depression, pain, and fatigue. The study reviewed multiple RCTs involving 475 cancer patients and found that VR interventions significantly improved anxiety levels (standardized mean difference = −2.07; 95% CI −3.81 to −0.34) and pain management (mean difference = −17.44; 95% CI −21.93 to −12.94) in patients undergoing chemotherapy. Three immersive VR scenarios (Table 1) that combined interactive tasks, visual stimuli, and biofeedback mechanisms produced greater symptom relief than noninteractive or less immersive VR interventions (*p* < 0.01; Garrett et al., 2024).^41^ As Figure 3 exemplifies, the combination of settings and variety of interactions in simulated environments, both through 3-dimensionally rendered videos and 3-dimensionally crafted gamified simulations, provide a sense of presence and engagement, distraction from pain, relaxation, and alleviating feelings of isolation.^42–43^

Furthermore, VR can facilitate social interactions and community building. Multiuser virtual environments remain uncommon in health care-based applications; however, one study exemplified this possibility, allowing individuals to interact in real time, promoting social connections and reducing feelings of loneliness by 25%.^44–46^ These environments can be particularly beneficial for those who are homebound or have limited mobility.^40,47^ Additionally, VR can be used to deliver self-management programs and educational interventions, enabling individuals to take control of their pain management and improve their psychological well-being, as in the case of the carefully designed multimodal and multi-session program RelieveVRx and EaseVRx (Table 1).^43,48,49^ In a study exploring the use of RelieveVRx with 1067, significant pain intensity and pain interference reductions were achieved through the use of a multimodal 8-week self-administered program.^48^ The diverse combination of modalities (Table 1) demonstrates how combining varying modalities for the complex biopsychosocial needs of chronic pain patients can lead to effective nonpharmacological alternative treatments. Furthermore, the long-term effects of such a VR software design were corroborated with a 12-month follow-up study demonstrating, along with complementary studies on EaseVRx, that multimodal software design leads to lasting effects for chronic pain patients and should be further explored for other complex conditions with biopsychosocial needs.^50^ As shown by Figure 3, this targeted integration of clinically designed software combining multimodal intervention techniques appears to provide lasting behavioral changes in the target population in self-administered settings.

One study highlighted that demographic predictors play a significant role in accessing pain care, and VR can serve as a valuable alternative for effective pain management, particularly for isolated individuals with limited access to traditional care. Participants from diverse backgrounds, including older adults, lower socioeconomic status individuals, or and racial/ethnic minorities, reported significant reductions in pain intensity (mean reduction of 1.9 points for age <65 and 1.9 for age 65+; both *p* < 0.001) and pain interference (mean reduction of 2.2 points for Caucasians, 2.3 for African Americans, and 2.3 for others; *p* < 0.001). The usability of the VR device received the highest rating (A+) on the System Usability Scale across all demographic groups, indicating its broad applicability and potential to overcome existing disparities in pain care access by providing a consistent, in-home, and low-barrier treatment option.^50^

### Chronic pain management using VR

VR has demonstrated benefits in modulating pain through various mechanisms, ranging from simple distraction to complex immersive experiences that capture attention, regulate stress, and activate higher cognitive functions by creating a sense of presence in an alternative reality.^51–54,108^ VR engages the psycho-neuro-endocrine-immunological system, aiding in emotional regulation, pain management, and potentially encouraging prosocial behaviors at a molecular level.^55–56^

VR interventions offer multidimensional benefits for chronic pain management by addressing psychosocial factors and enhancing pain relief through varying levels of immersion, distraction, and interactivity through sequentially designed experiences, as shown in Figure 3. A study by Morales Tejera et al. using Fulldive VR a more interactive and evolutive experience and VR Ocean Aquarium a simple video and distraction-based experience (Table 1) found that VR therapy significantly reduced kinesiophobia (*p* = 0.02, *d* = 0.67) and anxiety (*p* < 0.01, *d* = 0.54–0.82) in patients with nonspecific chronic neck pain compared with an exercise group.^47^ Virtual embodiment, involving sensory-motor capabilities and virtual body ownership, is crucial for enhancing VR effectiveness.^57^ Figure 3 exemplifies this by demonstrating how, during the activation phase, integrating fully embodied sensory-motor capabilities that align with the demographic and condition-specific needs of a population, they may retain learnings more effectively and therefore develop more effective lasting coping skills beyond the momentary reduction in symptoms. However, individual responses vary based on pain chronicity, psychological comorbidities, and prior VR exposure.^58–60^ Strong embodiment and presence through body ownership illusions and multisensory integration are essential for effective chronic pain management.^61–63^

A multimodal approach combining VR with transcranial direct current stimulation (VR-tDCS) has shown effectiveness in managing neuropathic pain in patients with spinal cord injury, with significant reductions in pain intensity maintained through follow-up.^64–66^ VR-tDCS promotes neuroplasticity and pain tolerance by realigning somatosensory and motor cortices, enhanced by minimizing visuo-proprioceptive incongruencies.^51,57,61^ Interactive VR interventions, such as virtual object manipulation and emotional state adaptation through visual and auditory feedback, can enhance therapeutic effects by engaging the brain’s sensory and motor pathways.^67,68^ A comparative study using Snow Canyon, an automated narrative-based distraction environment (Table 1), found that immersive VR achieved better outcomes than semi-immersive VR across all pain intensities, working consistently across demographic groups and helping those prone to catastrophizing pain.^68^ As Figure 3 shows, it is by building varying degrees of engagement in complementary environments that align with the target outcomes that allows VR software to achieve such impacts. Depending on the desire to alleviate momentary symptoms or achieve long-term outcomes, the degree of immersion, distraction, interactions, and engagement, as well as 3D visual environment design details, play a key role.

The importance of immersion was further demonstrated in venipuncture patients using the highly engaging and interactive multimodal Bear Blast experience (Table 1), where high VR immersion reduced postprocedural anxiety by 47% (*p* = 0.013), particularly in high anxiety sensitivity patients.^69^ These findings are supported by research in cancer patients receiving chemotherapy, where VR significantly reduced anxiety and fatigue, with high patient satisfaction and minimal cybersickness.^70^ A double-blind, randomized, placebo-controlled trial of EaseVRx (Table 1) demonstrated a 42.8% reduction in pain intensity and 51.6% reduction in pain interference, with 65% achieving clinically significant pain reduction compared with 40% in Sham VR.^49^ These short-term benefits were further validated by a 24-month study where a skills-based VR program incorporating cognitive behavioral therapy (CBT), mindfulness, and pain neuroscience education achieved a 24% reduction in pain intensity and 45% reduction in pain interference, compared with just 12% and 21%, respectively, in the Sham VR group.^48^

To enhance the effectiveness of VR therapy, it is essential to determine the optimal level of immersion required for different types of pain control. Research suggests that activating multiple senses—visual, auditory, and even tactile feedback—can significantly improve analgesic outcomes as exemplified by Figure 3.^71,72^ Simple effects such as engaging in conversation may have notable effects on pain perception, and careful attention to how individuals interact with and discover their environment and the emotions it triggers already has impacts on pain perception.^50,73^ Establishing guidelines that specify which sensory modalities to engage based on the type and severity of pain could make VR interventions more impactful. For example, acute pain might be effectively managed with high visual and auditory immersion, even adding tactile and olfactory stimuli to achieve optimal results; while chronic pain could benefit from multi-session, conversational or educational content designed for pain management.^41^

These findings highlight the importance of carefully crafting experiences from tutorials and onboarding that appropriately set expectations for patients, interactions in the environment that are physically manageable for patients while eliciting an emotional response through storytelling and educational content all the while incorporating real-time physiological feedback, personalized content, and interactive elements in VR design each being important elements exemplified by Figure 3.^74^ The advent of generative artificial intelligence (AI) creates unprecedented opportunities to develop dynamic, responsive therapeutic spaces that can adapt to individual patient needs while maintaining cost-effectiveness and scalability. As a patient goes through an experience, its level of engagement, educational complexity, and nature of interactions may become automated to adapt to the patient’s needs and the clinically desired outcomes (Figure 3). This AI-driven approach enables rapid iteration and personalization of VR interventions, ensuring they evolve with patient requirements while upholding ethical validity. It affords a new set of opportunities to mimic social or group interactions that have proven effective in supporting the mental health of patients.^75–78^ By leveraging generative AI, health care providers can create immersive, tailored VR distracting, relaxing, social or educational experiences that meet the unique needs of patients and care teams at scale, potentially revolutionizing the future of chronic pain management.^73^

### VR for loneliness and social isolation

VR technology and access to the internet can cultivate social connectivity and further promote social engagement.^79–80^ VR is increasingly recognized as a valuable therapeutic tool for managing pain and mitigating social isolation, especially among older adults and individuals with chronic illnesses or limited mobility. VR addresses these challenges by creating immersive experiences that distract from pain or help relax, educate, stimulate, and facilitate social interaction.^81–83^

During the COVID-19 pandemic, technology played a key role in education, recreating the social engagements of a physical classroom online.^84^ However, VR has been shown to offer a more immersive alternative to online socialization.^85^ Social VR environments build on traditional virtual worlds, such as Second Life, RuneScape, and Club Penguin, but enhance immersion through head-mounted displays, facilitating novel forms of embodied interaction.^86^

Social VR encompasses diverse forms of virtual interaction, ranging from avatar based to nonavatar, with both real people and nonplayable characters (NPCs), at varying levels of interactivity. Recent innovations include real-motion avatars that mirror users’ physical movements for enhanced social engagement.^46^ Understanding how these different interaction elements impact social connection, loneliness, and immersion in clinical settings is crucial, particularly for applications such as pain management.

An example is the Cedars-Sinai study of eXtended-reality Artificial Intelligence Assistant (XAIA), a platform combining VR with GPT-4-powered AI therapy to address therapist shortages. This system immerses users in virtual nature environments where they interact with an AI therapist, represented as a robot avatar, with built-in safety protocols for screening AI responses (Table 1). Testing with 14 participants experiencing mild-to-moderate anxiety or depression demonstrated XAIA’s safety and acceptability. Users valued the platform’s anonymity and calming environments, though some noted a preference for human therapists when seeking deeper therapeutic engagement. While promising as a supplemental mental health resource, especially for remote or homebound individuals, the study emphasized the need for further research to assess long-term clinical outcomes and enhance the AI’s therapeutic communication capabilities.^87^ Figure 3 shows how in the integration phase of a VR experience, the ability to reflect through conversationality, and identifying real-world applications of patients’ learnings may be key. During the engagement phase, it may facilitate understanding of key concepts and facilitate both satisfaction and engagement, rendering a far more pleasant and successful experience for the user. Much more work has to be done to ensure these interactions are clinically validated and made effective; however, they mark a significant step in bridging alternative solutions for access to care.

VR has also shown potential in supporting patients outside clinical settings, tracking self-reported and physiological markers for pain, loneliness, social isolation, and other predictors of mental health deterioration without stigmatizing pressures.^43^ Speech patterns can assist in diagnosing illnesses, and social VR can safely collect ecological momentary assessment data while tracking symptoms in a de-identified manner.^88^

Additionally, in one unique study using Vizard Software, an interactive, conversational avatar-based experience delivering VR-based CBT (Table 1) has shown mixed results. While it did not significantly reduce social avoidance in people with psychotic disorders, it helped with momentary anxiety and paranoid ideation, suggesting potential as an automated therapeutic alternative with operational benefits.^89^ VR’s potential to recreate social experiences through avatars and more complex levels of interactions, such as reliving memories in different contexts, presents intriguing opportunities.^85^ While studies have explored the quality of life in patients with chronic low back pain, some reported no significant improvement across multiple metrics. However, these studies found notable benefits in pain perception, highlighting the importance of experience design in achieving desired outcomes. The design of the experience here will play an important role, for example, gamified experiences with social connections may positively influence certain aspects of the pain experience, as suggested in Figure 3.^42^

Clinical outcomes from VR interventions for chronic pain management are promising. VR-based relaxation training reduced pain intensity by 33% and improved mood by 25% in patients with chronic pain.^43^ User satisfaction is also high, with 85% of participants finding VR interventions enjoyable and willing to recommend them to others.^44^ A randomized controlled pilot study found that 70% of participants experienced a clinically meaningful reduction in pain scores following VR interventions.^43^

## Discussion

This narrative review reveals a growing body of evidence supporting the role of VR in addressing the intersecting issues of chronic pain and loneliness. VR interventions present unique therapeutic solutions for managing chronic pain and associated loneliness and social isolation. Through immersive environments, VR provides both a distraction from pain and an opportunity for social engagement, alleviating the dual burden of physical and emotional chronic pain. Research also suggests that VR’s ability to enable social connections and offer pain relief is effective and adaptable across chronic conditions and patient demographics.

Significant improvements in psychological well-being and physical symptom management have been noted with VR interventions.^90–102^ Studies have shown that VR can reduce pain intensity, improve mood, and foster a sense of social connectedness by recreating interactive, virtual environments that simulate physical and social experiences otherwise inaccessible to many individuals with chronic pain.^59^ Additionally, integrating personalized, interactive elements within VR environments enhances engagement and strengthens therapeutic benefits.^92^ However, the degree of immersion required to achieve optimal outcomes varies. While more immersive VR environments often show greater efficacy in pain management and emotional well-being, studies have found that even with limited fidelity, significant improvements can be achieved.^68,93^ This suggests that increasing immersion and realism can be modulated depending on the level of pain and objective of the intervention. Therefore, highly lifelike environments and more reductivist environments may play different roles in modulating pain perception or social isolation.^69,94,95^ More studies exploring the difference between varying environment designs, levels of realism, and degree of interaction based on the nature of the condition are needed to arrive at a convincing conclusion. Studies often lack descriptions of the design of the software, whether it be for distraction, immersion, learning, or developing coping skills. Understanding how specific elements of design in the environment, as well as expectation setting through appropriate tutorials, play a key role in achieving results and are overlooked in publications. Leveraging a methodology like the one highlighted in Figure 3 may help make a step in that direction.^96^

Discrepancies were observed in VR’s effectiveness across different patient populations and conditions. Some studies found no significant improvements in long-term pain outcomes or mixed results for social engagement. This variability may be attributed to differences in VR environments, levels of immersion, types of headsets used, and the absence of standardized criteria for VR therapy.^97,98^ Without standardization, variations in hardware and software can significantly influence user experience and therapeutic outcomes. For instance, the degree of immersion provided by a VR headset can affect the user’s sense of presence in the virtual environment, thereby impacting the effectiveness of the intervention.^98^ These findings highlight the need to establish standardized protocols and VR intervention criteria. Developing guidelines that address hardware specifications, software design, and therapeutic content can ensure consistent outcomes. Additionally, designing VR therapies adaptable to individual needs and conditions is important, aligning with the broader literature on personalized medicine.

## Implications and Applications

VR presents significant practical implications for managing chronic pain and loneliness in health care settings, particularly for patients facing social isolation due to mobility limitations or chronic conditions. Its immersive social experience offers valuable integration opportunities in pain management clinics, rehabilitation programs, and mental health interventions, especially benefiting older adults struggling with social connections.

Stand-alone and self-guided VR interventions for pain management provide substantial financial advantages over traditional treatments that require the constant presence of a health care professional or costly long-term medication, frequent health care visits, and extensive resource utilization.^100^ By reducing reliance on pharmacological interventions and personnel oversight, VR can decrease direct medical costs, showing promise for other fields beyond pain management.^93,101^ This is particularly valuable in rural and underserved areas with health care professional shortages, where remote delivery through VR can complement existing telemedicine services by offering immersive therapeutic experiences that standard telehealth cannot provide, while similarly reducing travel costs and time commitments.^102,103^ VR’s scalability enables simultaneous treatment of multiple patients with low marginal costs after initial investment, contrasting with traditional therapies’ resource-intensive requirements.^71^ As VR technology becomes more accessible, long-term savings from reduced health care utilization can offset initial costs.^49,81^ However, widespread adoption requires addressing affordability, accessibility, and user experience challenges to realize VR’s full potential in improving patient outcomes.

## Practical Implications

While VR shows promise for managing chronic pain and loneliness, several challenges must be addressed for it to be effectively applied. Although consumer VR systems have become increasingly affordable compared with traditional resource-intensive treatments, high-quality VR systems with multiple sensors and high-resolution displays can still be expensive, potentially limiting access for some health care providers and patients.^104^ Additionally, VR systems require technical expertise for setup and maintenance, which can be a barrier for individuals lacking tech skills or support.^105^ Providing user-friendly technical support is crucial for broader adoption.

User experience also plays a critical role. Many users experience motion sickness or physical discomfort during VR sessions, especially those with chronic pain.^105^ Extended VR use can cause neck strain and eye fatigue.^106^ Future VR designs need to focus on ergonomics and minimizing discomfort. The efficacy and long-term effects of VR are also important. VR’s effectiveness can vary based on user engagement and the nature of the condition being treated, with some patients responding better than others.^91^ More research is needed to understand the long-term sustainability of VR’s benefits and any potential side effects.^107^

The therapeutic efficacy of VR depends on the degree to which interventions are personalizable and immersive. Chronic pain and loneliness are personal experiences, and one-size-fits-all solutions are unlikely to work. Customizable VR programs that adapt to individual needs are important for increasing outcomes.^107^ Ultimately, creating high-quality VR content requires substantial resources, yet it is fundamental to the success of these interventions. Regular updates based on user feedback and new research are required to maintain engagement and ensure the therapy remains effective.^107^

## Limitations and Considerations

This narrative review has several limitations. The literature’s limited focus on racial and ethnic minorities, with only one study examining demographic factors, constrains its applicability across diverse groups. In this study, Caucasian participants showed higher completion rates and device usability compared with African American participants in a VR chronic pain program.^50^ The review’s quality depends on the included studies’ limitations, particularly regarding VR immersion levels, which correlate with pain reduction outcomes. Key constraints in selected studies include heterogeneous patient populations, variations in VR equipment and session duration, and inconsistent cybersickness and user satisfaction reporting.^41^ These factors, combined with variable VR effectiveness across patient characteristics, emphasize the need for standardized intervention guidelines and individualized, user-centered design, though resource constraints challenge such tailored approaches.^96^ The need for consistent detail in the description of design, user interface, user experience, and implementation science involved in the creation of VR experiences may help identify a taxonomy of successful experiences at the intersection of fields and conditions.^96^

## Future Directions

Future developments in VR applications should focus on refining therapeutic environments, enhancing avatar realism, and improving social interaction design to maximize benefits across varied populations. The use of NPCs has the potential to expand the benefits of VR for social isolation, loneliness, and pain. As AI-based conversational agents become more integrated into VR development, they have the opportunity to extend the capabilities of VR in therapeutic settings, as shared storytelling and group therapy have shown to be highly effective for a multitude of conditions from cancer to chronic pain. Further clinical studies are also needed to better understand VR’s efficacy in different contexts and optimize its design to effectively meet therapeutic goals.

Improving accessibility is crucial for the broader adoption of VR in addressing chronic pain and loneliness. Reducing the cost of VR equipment and software, developing more affordable headsets, and leveraging advancements in mobile VR technology are essential steps. Providing user-friendly technical support can also help individuals with limited technical skills adopt the technology more efficiently.

Cultural adaptivity is also important for making VR accessible and effective across diverse populations. Future VR applications should incorporate culturally relevant content, languages, and contexts to resonate with users from different backgrounds. Collaborating with community members, health care providers, and cultural experts can help create inclusive VR experiences that address unique cultural needs related to pain management and social connection.

The role of interactive, immersive experiences in enhancing VR’s therapeutic potential should be further explored. Research should examine how different levels and types of interactivity impact pain perception, social engagement, and overall well-being. Identifying the optimal balance of interactivity and immersion for various patient populations can enable more precisely tailored VR interventions.

AI offers promising opportunities to create personalized, adaptive VR experiences. AI-powered virtual agents could provide empathetic social support, guide users through pain management techniques, or adjust stimuli based on biometric data. As AI advances, it may enable the development of socially and emotionally intelligent VR companions that offer targeted support to individuals dealing with chronic pain and isolation. As Figure 3 highlighted, crafting onboarding experiences that adapt to the demographic needs of patients, adapting environments for didactic learning, level of interaction for practicing skills-based learning, conversationality for integration, understanding, and reflection all play a part in the design of successful immersive experiences. Moreover, moving beyond the short-term effects and looking to the effects of VR in helping being about behavioral shifts shows significant promise.

Integrating VR into collaborative health care teams and comprehensive treatment plans is essential for maximizing its impact on chronic pain management and social determinants of health. Transdisciplinary collaboration among health care providers, technologists, researchers, and patient advocates should focus on empowering patients, unburdening care teams, addressing social determinants, and enhancing treatment outcomes. VR interventions should be designed to increase patients’ self-efficacy, provide valuable data insights to optimize care delivery, and complement other evidence-based therapies. Health care organizations and policymakers should invest in training, research, and infrastructure to support the integration of VR into coordinated care models.

Longitudinal studies are needed to assess the long-term efficacy and safety of VR interventions. More RCTs comparing VR to traditional treatments and other digital health tools are necessary to establish its comparative effectiveness and identify best practices for implementation. Tracking outcomes over extended periods is important to determine if VR’s benefits are sustained and monitor for potential adverse effects.

## Conclusions

This review demonstrates the potential of VR interventions in addressing the interconnected challenges of chronic pain and loneliness. Current evidence indicates that VR-based therapies can effectively reduce pain intensity while simultaneously providing opportunities for social engagement, which is particularly valuable for individuals with limited mobility or access to traditional care settings. The integration of advanced features, such as AI-enhanced therapeutic environments and real-time biofeedback mechanisms, shows promise for delivering personalized interventions that address both the physical and psychosocial dimensions of chronic pain management.

Despite these developments, several limitations must be addressed before widespread implementation. These include the need for standardized intervention protocols, long-term effectiveness data beyond 24 months, and solutions to accessibility barriers, particularly among underserved populations. Future research should focus on establishing consistent outcome measures, clearly defining the nature of the software use, characteristics of the environments, type of experience, and modality used. It should also investigate cost-effectiveness and realities of implementation in medical contexts compared with traditional treatments to help ensure accessibility of the solution, often impeded by poor integration into care workflows. This will help in developing scalable solutions that can be integrated into existing health care frameworks. Addressing these challenges will be essential for transforming VR from a promising technology into a standardized therapeutic tool for comprehensive chronic pain management to address loneliness and social isolation.
